# *Leishmania major* Dihydrolipoyl dehydrogenase (DLD) is a key metabolic enzyme that drives parasite proliferation, pathology and host immune response

**DOI:** 10.1371/journal.ppat.1012978

**Published:** 2025-03-17

**Authors:** Somtochukwu S. Onwah, Zhirong Mou, Gaurav Gupta, Patience Obi, Nnamdi Ikeogu, Ping Jia, Wen-Wei Zhang, Saeid Ghavami, Ayesha Saleem, Jude Uzonna

**Affiliations:** 1 Department of Immunology, Rady Faculty of Health Sciences, University of Manitoba, Winnipeg, Manitoba, Canada; 2 Faculty of Kinesiology and Recreation Management, University of Manitoba, Winnipeg, Manitoba, Canada; 3 Department of Microbiology and Immunology, McGill University, Montreal, Quebec, Canada; 4 Department of Human Anatomy and Cell Science, Rady Faculty of Health Sciences, University of Manitoba, Winnipeg, Manitoba, Canada; 5 Department of Medical Microbiology and Infectious Diseases, Rady Faculty of Health Sciences, University of Manitoba, Winnipeg, Manitoba, Canada; 6 Department of Pathology, Rady Faculty of Health Sciences, University of Manitoba, Winnipeg, Manitoba, Canada; UT Southwestern: The University of Texas Southwestern Medical Center, UNITED STATES OF AMERICA

## Abstract

Identifying antigens that elicit protective immunity is pivotal for developing effective vaccines and therapeutics against cutaneous leishmaniasis. Dihydrolipoyl dehydrogenase (DLD), a mitochondrial enzyme involved in oxidizing lipoamides to facilitate electron transfer for energy production and metabolism, plays a critical role in virulence of fungi and bacteria. However, its function in *Leishmania* virulence and pathogenesis remains unexplored. Using a CRISPR-Cas9-based approach, we generated DLD-deficient *Leishmania (L.) major* parasites and a complementary add-back strain by episomally reintroducing DLD gene into the knockout parasites. Loss of DLD significantly impaired parasite proliferation in axenic cultures and infected macrophages compared to wild-type (WT) and add-back control parasites. These defects were linked to reduced ROS production, impaired mitochondrial permeability, an enhanced oxygen consumption rate, and alterations in mitochondrial ultrastructure. In murine models, DLD-deficient parasites failed to cause observable lesions and exhibited significantly reduced parasite burdens compared to WT and add-back control strains. Notably, mice infected with DLD-deficient parasites displayed blunted immune responses compared to their WT controls. Importantly, vaccination with DLD-deficient parasites conferred robust protection against virulent *L. major* challenge, characterized by a strong IFN-γ-mediated immune response. These findings establish DLD as an essential metabolic enzyme for *L. major* intracellular survival and pathogenesis. Targeting DLD not only impairs parasite viability but also holds promise as a novel strategy for vaccine development to combat cutaneous leishmaniasis.

## Author summary

Vaccination against cutaneous leishmaniasis is possible, because individuals who recover from natural disease acquire lifelong immunity. However, no approved vaccine for humans currently exists. Developing a vaccine requires identifying antigens capable of inducing a long-lasting protective response during *Leishmania* infection. One potential target is Dihydrolipoyl dehydrogenase (DLD), an enzyme essential for the parasite’s energy production.

Here, we used the CRISPR-Cas9 gene-editing tool to delete DLD gene from *Leishmania major*. Additionally, we created a control strain by reintroducing DLD gene into DLD-deficient parasites. The absence of DLD impaired proliferation of *L. major* in culture and within macrophages and compromised mitochondrial function. In mice, the DLD-deficient parasites caused no disease pathology but altered the host immune response. Significantly, mice vaccinated with the DLD-deficient parasites developed strong immunity against a virulent *L. major* challenge. These findings highlight the critical role of the DLD gene in parasite survival and its potential as a target for vaccine development.

## Introduction

Leishmaniasis is a disease caused by protozoan parasites of the genus *Leishmania*. It is transmitted through the bite of an infected sandflies during blood meals. The disease is prevalent in regions across Europe, Asia, America and Africa [1,2]. According to the World Health Organization (WHO), an estimated 12 million cases exist globally, with 1.5-2 million new cases reported annually [3]. The parasite primarily infects vertebrate hosts such as rodents, canids and humans [4]. Once injected into the mammalian host, the promastigotes are phagocytosed by macrophages, where they transform and differentiate into amastigotes, which proliferate within parasitophorous vacuoles (PVs) [1,2].

Leishmaniasis presents with a range of clinical manifestations depending on the Leishmania species involved. These can vary from cutaneous leishmaniasis, characterized by formation of crusted nodular or ulcerative lesions that typically heal spontaneously within 3–18 months [1,2], to visceral leishmaniasis (VL), a severe form affecting the liver, spleen and bone marrow, which is fatal if left untreated [1,2].

Resistance against *Leishmania* infections is associated with the induction of a robust Th1 immune response. This response triggers the release of IFN-γ from CD4^+^ T cells, which activates *Leishmania*-infected macrophages to mediate nitric oxide-dependent intracellular parasite killing [4,5]. In contrast, failure to mount an effective Th1 immune response is associated with chronic and severe infections [1,2]. In such cases, IL-4 and IL-13, produced by Th2 cells, promote alternative activation of macrophages that inhibits nitric oxide release, thereby preventing effective parasite killing [4,5]. In addition, these cytokines suppress classical macrophage activation and enhance polyamines synthesis, creating a permissive environment for parasite proliferation [4,5].

Changes in the activity of different metabolic enzymes enable *Leishmania* parasites to persist and proliferate within the harsh environmental conditions of the parasitophorous vacuoles (PVs) that is highly acidic and poor in glucose [6,7]. Indeed, metabolic enzymes are critical for supporting the stringent metabolic adaptations observed with amastigotes in the PVs [8,9]. Amastigote stages of *Leishmania* rely on fatty acids and amino acids to produce metabolic energy, which is solely driven by the Tricarboxylic acid (TCA) cycle. This process is essential for maintaining pH and homeostasis [10]. A key component of this cycle is the pyruvate dehydrogenase complex, which comprises of pyruvate dehydrogenase, lipoyl transacetylase, and dihydrolipoyl dehydrogenase (DLD), which catalyzes the production of metabolites crucial for energy metabolism [11]. Recently, we showed that naturally processed peptides derived from DLD of *L. major* are presented by dendritic cells to CD4^+^ T cells in infected animals [12]. These DLD-specific CD4^+^ T cells expand and produced effector cytokines, such as IFN-γ and TNF, upon *L. major* infection in C57BL/6 mice [12]. Given the stringent metabolic demands faced by amastigotes in the PV, we reasoned that targeting their metabolic and energy requirements could offer valuable insights for developing novel therapies against the parasite [13].

Several *Leishmania* strains deficient in key metabolic enzymes such as those involved for gluconeogenesis [14], iron uptake [15], purine metabolism [16] and glycoconjugate synthesis [17], have been shown to survive within their macrophage hosts by establishing low-level chronic infection without causing severe pathology [14–17]. In line with this observation, we previously showed that the gluconeogenesis enzyme, phosphoenolpyruvate carboxykinase (PEPCK), is critical for virulence of *L. major* [18]. Mice infected with PEPCK deficient *L. major* did not develop any lesion and harbored significantly less parasite load compared to their wild-type (WT) infected controls, suggesting a loss of virulence [18]. In other pathogens, such as *Mycoplasma gallisepticum, Streptococcus pneumoniae*, and *Mycobacterium tuberculosis* DLD has been shown to be a critical virulence factor [8,19–21]. Similarly, in the closely related parasite, *Trypanosoma brucei*, the absence of DLD in the blood stream form prevents establishment of infection [22]. However, the impact of DLD deficiency in *Leishmania* virulence and host immune response has not yet been investigated.

The current study investigated the role of DLD in the virulence of *Leishmania major* and its impact on the host immune response. Using genetic manipulation, we successfully generated *DLD* gene deficient *L. major* along with their complementary addback controls. Our findings revealed that DLD deficient parasites exhibited impaired in proliferation both *in vitro* and *in vivo.* These parasites persist in infected animals without causing significant pathology and protected mice against virulent *L. major* challenge. We further demonstrated that DLD is a critical mitochondrial enzyme involved in regulating oxidative phosphorylation in parasites and is important for their intracellular survival in infected host cells.

## Results

### Confirmation of DLD deletion in *L. major*

**Fig 1:**
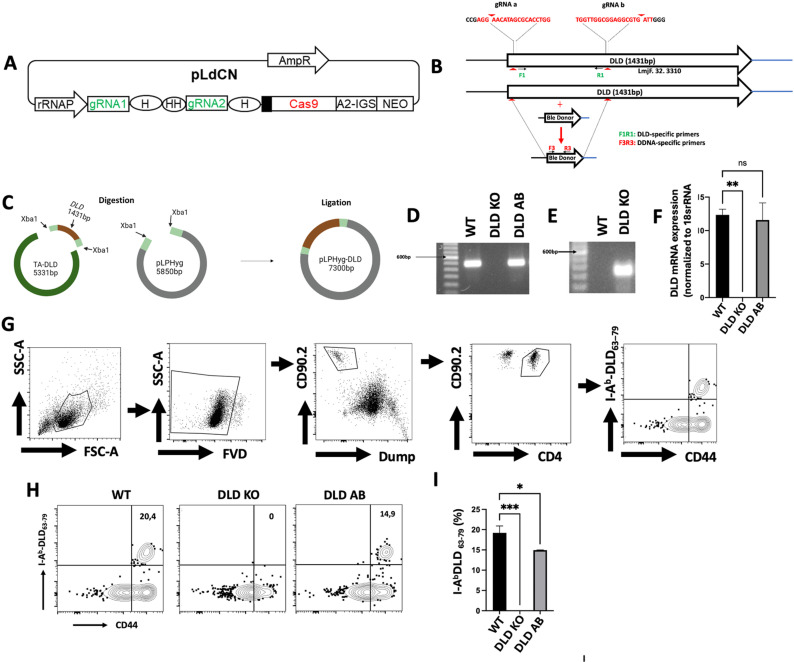
Deletion strategy and confirmation of *DLD* gene deletion in *Leishmania major.* Schematic of the all-in-one plasmid, pLdCN where gRNA is inserted under the control of the RNA ribosomal promoter (A). Schematic description of *Leishmania* all-in-one CRISPR plasmid (pLdCN) expressing dual gRNAs targeting the *L. major* DLD gene flanking sequences (B) and the strategy used to generate the complementary Add Back strains (C). Qualitative (D) and quantitative (F) PCR analyses showed the absence of DLD amplicon and mRNA expression in *L. major* DLD null mutant (DLD KO) and the restoration of DLD in the complementary addback controls (DLD AB). PCR analysis showing the presence of Bleomycin resistance gene cassette in the DLD KO but not in the WT *L. major* (E). Flow cytometry gating strategy for detecting DLD-specific CD4^+^ T cells in the spleen and dLN of mice infected with WT, DLD KO or DLD AB (G). Confirmation of DLD deficiency in DLD KO parasites by assessing the percentages (H & I) of DLD-specific CD4^+^ T cells in C57BL/6 mice infected with WT, DLD KO and DLD complementary addback (AB) control parasites using DLD tetramer after 5 weeks post-infection. Results presented in H & I are representative of 2 independent sets of experiments (n = 3-4 mice per group) with similar results, *, p < 0.05; **, p < 0.01; ***, p < 0.001; ns, not significant.

Using proteomics and reverse immunology, we previously identified an immunodominant peptide from *Leishmania major* DLD (DLD_63-79_) that induced strong CD4^+^ T cell response in infected mice [12]. Vaccination with full length DLD or DLD_63-79_ with CpG adjuvant induced robust protective immunity against virulent wild-type *L. major* challenge [12], indicating that *Leishmania* DLD modulates host immune response. To clearly understand the impact of DLD on parasite virulence and host immune response, we deleted the *DLD* gene (GCVL-2 DLD on chromosome 32 but a GCVL-1 DLD isoform is present in Chromosome 29) using CRISPR/Cas9 with a dual gRNA in pLdCN vector (Fig 1A) that ensures cleavage within two regions of the gene (Fig 1B). A bleomycin resistance cassette was introduced at the cleavage site through homology-directed repair to minimize error-prone DNA repair (Fig 1B) [23]. Additionally, we generated a DLD Add Back (AB) parasite line by reintroducing the full-length *DLD* gene into the DLD KO line using the pLPHyg-DLD plasmid, ensuring that the observed phenotypes were not due to CRISPR/Cas9-related mismatches (Fig 1C). We confirmed *DLD* gene deletion in the knockout strains (Fig 1D) and restoration of *DLD* gene in the complemented AB controls (Fig 1D). The presence of the Bleomycin resistance gene in the DLD KO parasites but not their WT controls (Fig 1E) confirmed successful homology-directed repair (Fig 1E), and absence of DLD mRNA expression in the DLD KO parasites and its restoration in DLD AB parasites further confirmed deletion of *DLD* gene in the KO parasites (Fig 1F). Interestingly, a western blot analysis detected a DLD band in the DLD knockout (KO) (S1A Fig). This likely occurred due to the use of a polyclonal anti-DLD primary antibody, which may recognize epitopes shared by both the GCVL-1 and GCVL-2 products. The close molecular weights of these isoforms (56 kDa for GCVL-1 and 51 kDa for GCVL-2) make it challenging to distinguish between them on a western blot. We further confirmed the specificity of our CRISPR-Cas9 system by showing that the absence of GCVL-2 DLD in the KO parasite did not affect the expression of GCVL-1 DLD or its mRNA (S1B and S1C Fig).

To further confirm *DLD* gene deletion in DLD KO parasites, we used an *in vivo* approach. We had previously generated DLD_63-79_ tetramers that reliably detect DLD-specific CD4^+^ T cells in *L. major*-infected mice [12]. We hypothesized that no expansion of DLD-specific CD4^+^ T cells would occur in mice infected with DLD KO *L. major*. Using the prescribed gating strategy (Fig 1G), infection with WT *L. major* resulted in a significant expansion of DLD-specific CD4^+^ T cells in the spleens and dLNs at 5 weeks post-infection. In contrast, DLD-specific CD4^+^ T cells were undetectable in mice infected with DLD KO parasites (Fig 1H and 1I). These results confirm the successful deletion of *DLD* gene in *L. major*.

### DLD deficiency in *L. major* results in impaired proliferation in axenic cultures and inside macrophages

**Fig 2:**
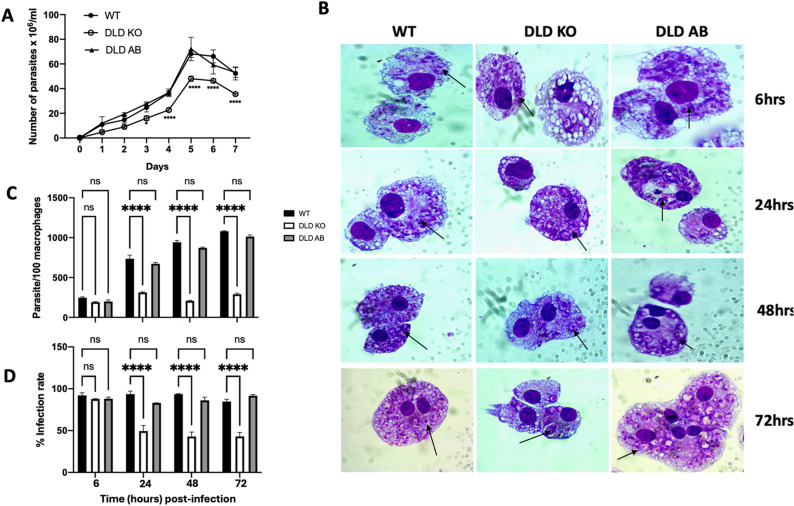
Proliferation of DLD deficient *Leishmania major* is impaired proliferation in axenic culture and macrophages. Equal numbers of WT, DLD KO and DLD AB *L. major* were cultured in axenic culture and counted daily using a hemocytometer (A). Bone marrow-derived macrophages were infected with WT, DLD KO and DLD AB *L. major* at a 10:1 parasite to cell ratio. After 6 hours, uninfected parasites were washed off, and at the indicated times, the infection rate was monitored by staining cytospin preparations with Giemsa stain and viewing under a light microscope (B). Macrophage infection rate was further quantified by estimating the number of parasites per 100 macrophages (C) and percentage of infected cells (D). Black arrows indicate amastigotes. Results presented are representative of 2 independent sets of experiments with similar results. ****, p < 0.0001; ns, not significant.

Impaired parasite proliferation is often associated with a reduced virulence and/or infectivity [24]. Although DLD is known to be a critical mitochondrial enzyme [11,25], whether its role in *L. major* proliferation is unknown. We found that, compared to the WT or DLD AB control groups, DLD KO parasites exhibited significantly impaired proliferation in axenic culture, particularly at the peak of the logarithmic growth phase, which extended into the stationary growth phase (Fig 2A).

**Fig 3:**
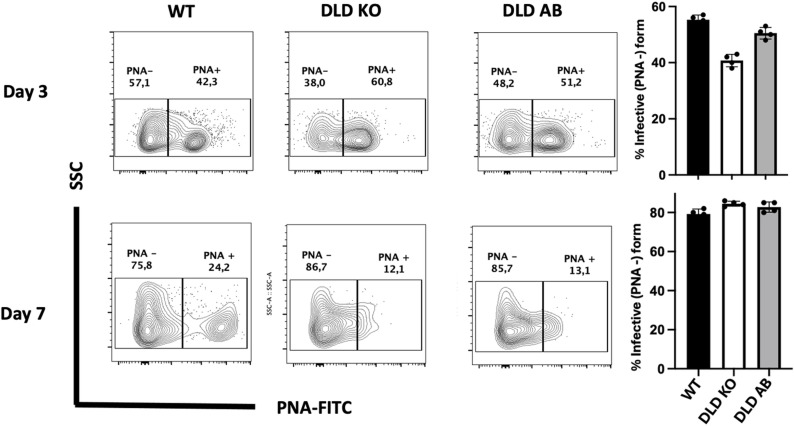
Deletion of *DLD* gene in *L. major* does not impact their ability to differentiate into metacyclic promastigotes in axenic culture. WT, DLD KO and DLD AB *L. major* at logarithmic (day 3) and stationary (day 7) phases in axenic cultures were stained with FITC-conjugated PNA and the uptake of PNA was determined by flow cytometry. Results presented represent 2 independent sets of experiments with similar results.

We investigated whether the deletion of DLD gene affects the ability of *L. major* to transform into the infective promastigote form in axenic culture [26]. To do this, we compared the binding ability of day 3 and day 7 WT and DLD deficient parasites to PNA, which binds strongly to procyclic promastigotes but not metacyclic promastigotes due to differences in lipophosphoglycan (lpg) sugar capping. At day 3 (logarithmic growth phase), the percentage of PNA-negative WT and DLD KO parasites was low but comparable between WT and DLD KO strains (Fig 3). By day 7, the percentage of PNA negative parasites increased significantly in both WT and knockout strains, consistent with their transformation into the infective promastigotes (Fig 3). These results suggest that *DLD* gene deletion does not affect their metacyclogenesis, and hence the ability to differentiate into their infective forms.

Macrophages are the primary host cell for the establishment, proliferation and spread of *Leishmania,* including dendritic cells and neutrophils [4,5,27]. Given the impaired proliferation of DLD KO parasites in axenic culture, we hypothesized that their proliferation within macrophages, their definitive host cells, will also be affected. As shown in Fig 2B, DLD KO parasites exhibited compromised proliferation inside infected BMDMs compared to their WT and DLD AB controls. This was manifested as reduction in the total number of parasites per 100 macrophages (Fig 2C) and lower infection rate (number of infected cells per 100 macrophages, Fig 2D). The impaired proliferation of DLD KO parasite was not due to defect in their ability infect macrophage, as there were no differences in the percent infection or number of parasites per 100 macrophages between DLD KO and WT parasites at 6 hours post infection (Fig 2C and 2D). Taken together, these results indicate that DLD is essential for the intracellular survival and proliferation of *L. major* in macrophages.

### DLD deficiency impacts mitochondrial function and metabolism in *L. major*

**Fig 4:**
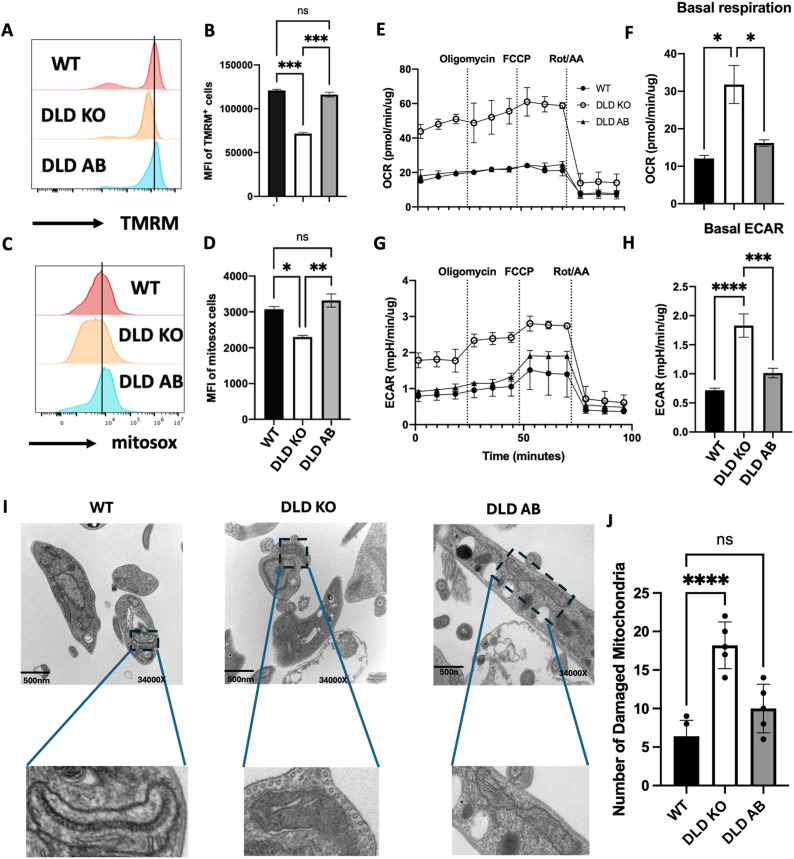
DLD deficiency in *L. major* impacts parasites mitochondrial metabolism and function. Histogram plots and mean fluorescent intensity of WT, DLD KO and DLD AB *L. major* stained with TMRM (A & B) and Mitosox (C & D) dyes. Five million (5 x 10^6^) WT, DLD KO and DLD AB *L. major* promastigotes were immobilized using poly-D-lysine and treated with Oligomycin, FCCP, and Rotenone/antimycin and mitochondrial stress was assessed using the XF24 analyzer in an XF supplemented media. Following normalization with parasite protein concentration, oxygen consumption rates over time were assessed (E) and basal respiration was calculated (F). The acidification of the media was assessed as extracellular acidification rate (ECAR) over time (G), and the basal levels were determined (H). The logarithmic growing parasites were fixed and visualized to reveal double membrane mitochondria using TEM (I). Images were quantified using a field-based approach (J). Images were taken at 34000X magnification. *, p < 0.05; **, p < 0.01; ***, p < 0.001; ****, p < 0.0001; ns, not significant.

Given that DLD is a critical mitochondrial enzyme for energy generation, we hypothesized that its absence would affect mitochondrial function in *L. major*. To test this, we assessed mitochondrial membrane potential using TMRM, a dye that penetrates and accumulates in healthy and functional mitochondria membrane [28]. DLD KO parasites showed a significant decrease in mitochondrial membrane potential compared to WT and DLD AB controls, suggesting compromised mitochondrial membrane integrity (Fig 4A and 4B). This impairment was accompanied by significant reduction in mitochondrial reactive oxygen species (ROS) levels compared controls (Fig 4C and 4D).

A decrease in mitochondrial membrane potential and ROS production suggests impaired mitochondrial respiration, which can be measured by the oxygen consumption rate (OCR, 36). To assess this, we exposed the different parasite lines to metabolic inhibitors (oligomycin, FCCP or rotenone/antimycin A), targeting oxidative pathways. Surprisingly, DLD KO parasites displayed higher oxygen consumption rate than WT and DLD AB controls (Fig 4E), with significantly increased basal OCR (Fig 4F). This suggests that the absence of *DLD* gene product forces the parasites to rely on increased oxygen to meet their energy needs. We also assessed glycolytic activity using extracellular acidification rate (ECAR), which is a measure of glycolytic flux [30]. We exposed the parasite lines to metabolic inhibitors (oligomycin FCCP, or rotenone/antimycin A). DLD KO parasites had higher ECAR compared to WT and DLD AB controls following treatment with these inhibitors (Fig 4G). Furthermore, the basal acidification rate in DLD cultures was significantly elevated before drug treatment (Fig 4H). These findings indicate that the loss of DLD in *L. major* disrupts mitochondrial respiration driving the parasite to compensate by increasing glycolytic flux to meet its energy needs.

The double membrane in mitochondria traps electrons critical for ATP production [31]. Given the energy deficit in DLD-deficient parasites, we examined their outer mitochondrial ultrastructure using transmission electron microscopy (TEM). In DLD KO parasites, their outer mitochondrial membrane appeared fragmented or swollen, while the inner membrane showed disorganized or absent cristae, indicating severe structural damage (Fig 4I). In contrast, WT and DLD AB parasites displayed intact double membranes and well-organized cristae (Fig 4I). Using a field-based assessment method, we observed significantly greater mitochondrial damage in DLD KO parasites compared to WT and DLD AB parasites (Fig 4I). These structural defects in mitochondria of DLD KO parasites align with functional impairments, including decreased mitochondrial membrane potential and ROS production, and provide direct evidence of mitochondrial stress and dysfunction in the absence of the DLD gene. This mitochondrial damage likely contributes to impaired proliferation inside macrophages *in vitro* and *in vivo*. Together, these findings underscore the critical role of DLD in maintaining mitochondrial integrity, function and parasite viability.

### DLD deficient *L. major* does not induce pathology in infected mice

**Fig 5:**
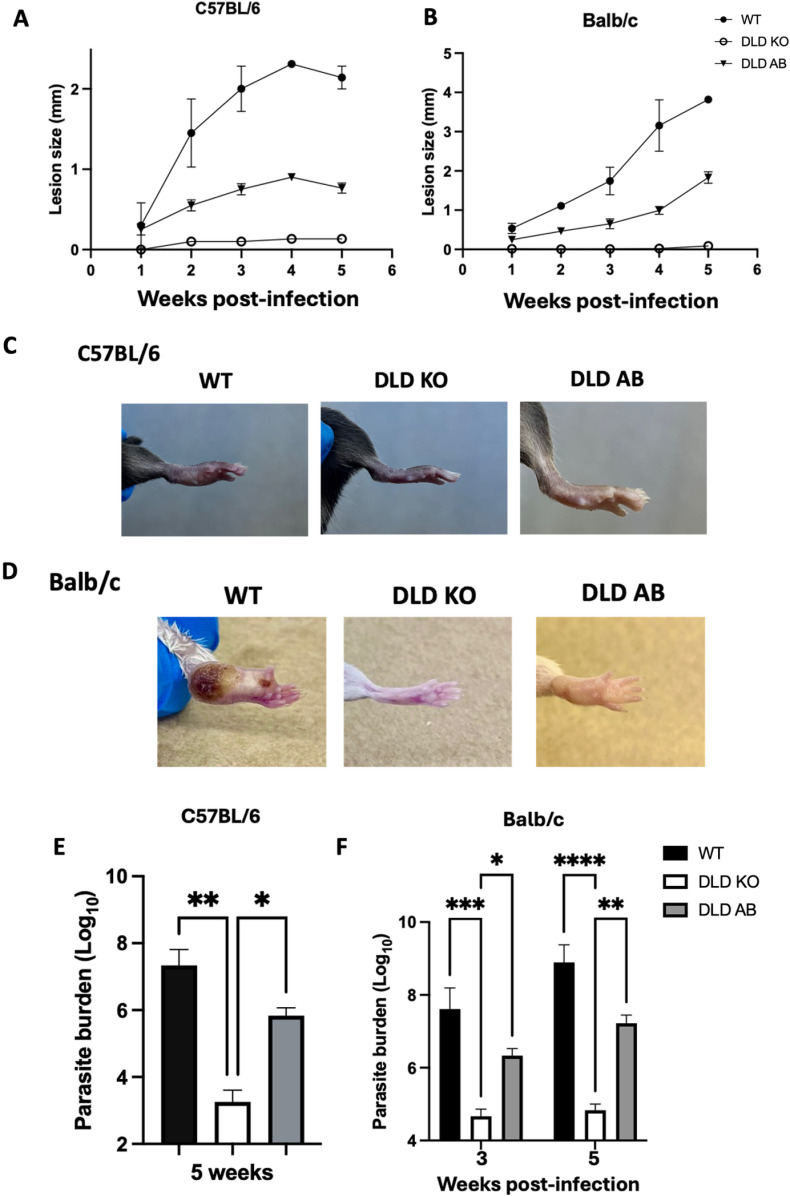
DLD deficient parasites are attenuated in vivo and do not cause lesion in mice. Six to eight (6–8) weeks old C57BL/6 (A, C and E) and Balb/c (B, D and F) mice were infected with 1 x 10^6^ WT, DLD KO and DLD AB *L. major* in their left hind footpads and the development and progression of lesion was monitored weekly with digital calipers. At 5 weeks post-infection (when lesion sizes in the groups infected with WT parasites reached the humane endpoint for the Balb/c mice), the animals were sacrificed, and pictures of lesions were taken (C & D), and the parasite burden was determined by limiting dilution (E & F). The results presented are representative of 3 independent sets of experiments (n = 6-8 mice per group) with similar results. *, p < 0.05; **, p < 0.01; ***, p < 0.001; ****, p < 0.0001.

Previous studies have shown that DLD played critical roles in virulence of *Streptococcus pneumonia* [20] and *Mycobacterium tuberculosis* [21] *in vivo*. Given impaired proliferation of DLD KO parasites *in vitro*, we hypothesized that their virulence would also be compromised *in vivo*. Indeed, C57BL/6 and Balb/c mice infected with DLD KO *L. major* did not develop any observable footpad lesions up to 5 weeks post-infection unlike those infected with WT and DLD AB control parasites (Fig 5A-D). This lack of footpad lesion was associated with significant reduction in parasite burden in DLD KO infected mice compared to WT or DLD AB controls (Fig 5E and 5F). Interestingly, complementation of DLD KO parasites with an episomal *DLD* gene only partially restored lesion development and parasite burden (Fig 5B and 5C), consistent with previous studies on unrelated genes which showed that episomal re-expression of a CRISPR-induced gene deletion does not fully recapitulate WT phenotype [32]. To investigate whether the incomplete restoration of the WT phenotype in DLD AB-infected animals was due to the loss of hygromycin selection pressure *in vivo*, we assessed GCVL-2 DLD expression in DLD AB parasites cultured with or without hygromycin for up to 8 weeks. In cultures without hygromycin, we observed a decline in DLD expression (both PCR and RT-PCR), beginning around 7 days and resulting in a complete loss by 8 weeks (S2 Fig). These findings align with previous studies indicating that continuous antibiotic selection pressure is essential to maintain *Leishmania* genes carried on the pLPHyg episomal vector [33]. Despite this limitation, episomal gene expression delivery methods remains a versatile method for stable gene delivery in *Leishmania* [34]. Since DLD mRNA expression and proliferation were restored in DLD KO, the observed phenotypes in DLD KO parasites are not due to off target CRISPR effects. Interestingly, dose-response experiments revealed that higher inoculum doses (5–10 × 10⁶ parasites) of DLD KO parasites led to lesion development (S3A Fig) and increased parasite burdens (S3B Fig). However, these were still significantly lower than those caused by 1 × 10⁶ WT parasites. These findings further underscore the critical role of DLD as an enzyme regulating *Leishmania* proliferation and lesion progression in infected mice.

### Infection with DLD deficient *L. major* results in blunted host immune response

**Fig 6:**
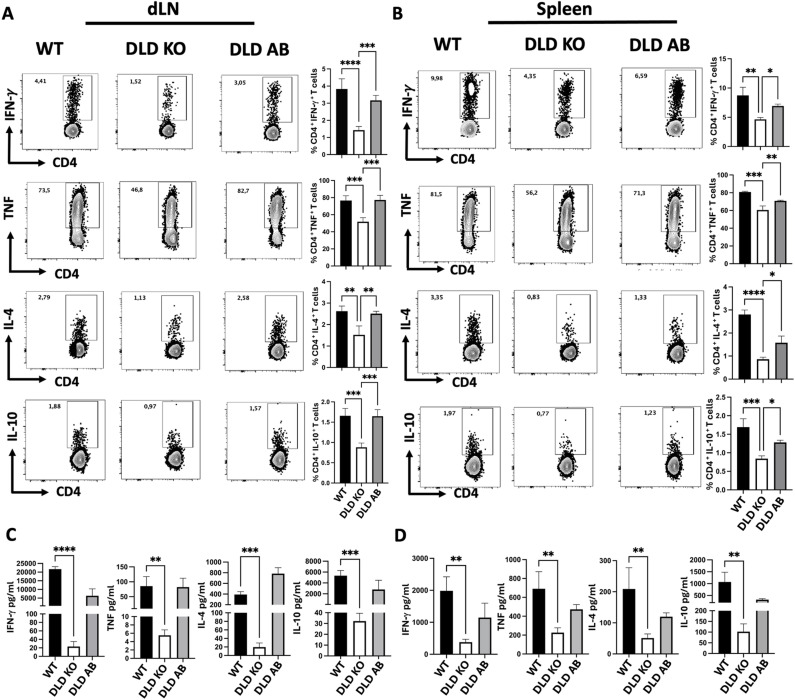
Deficiency of DLD in *Leishmania major* results in blunted host immune response. Six to 8 weeks old Balb/c mice (6-8 mice per group) were infected with 1 x 10^6^ WT, DLD KO and DLD AB *L. major*. At 5 weeks post-infection mice were sacrificed and single-cell suspensions from draining lymph nodes (dLNs) and spleens were stimulated with PMA/ionomycin and brefeldin cocktail directly *ex vivo* for 4 hours and the proportion of cytokine (IL-4, IL-10, TNF and IFN-γ)-producing CD4^+^ T cells (A & B) were determined by flow cytometry. Some cells were restimulated with soluble *Leishmania* antigen (SLA, 50 μg/ml) for 72 hours and the levels of cytokine in the culture supernatant fluids were assessed by ELISA (C & D). Results presented are representative of 3 independent sets of experiments (n = 6-8 mice per group) with similar results. *, p < 0.05; **, p < 0.01; ***, p < 0.001.

Resistance to *Leishmania major* infection (effective resolution of cutaneous lesion and clearance of the parasites) is linked to a strong IFN-γ response [4,5], whereas susceptibility correlates with the production of macrophage deactivating cytokines such as IL-4, IL-13 and IL-10 [4,5]. Since we observed no lesion development and significantly reduced parasite burden in mice infected with DLD KO parasites (Fig 5), we investigated whether this was due to an altered immune response. We analyzed the frequency of cytokine-producing CD4^+^ T cells from the spleen and dLN of mice infected with DLD KO parasites directly *ex vivo*. Compared to WT or DLD AB controls, mice infected with DLD KO parasite showed significantly lower frequencies of CD4^+^ T cells producing IFN-γ, IL-4, IL-10 and TNF-α in both the spleen and draining lymph node, dLN (Fig 6A and 6B). Similarly, supernatant from the spleen and draining lymph node cell cultures from mice infected with DLD KO parasites contained significantly lower levels of IL-4, IFN-γ, IL-10 and TNF-α in the culture supernatant fluids compared to those infected with WT and DLD AB control groups (Fig 6C and 6D). These results indicate that DLD deficiency in *L. major* leads to reduced host immune response in mice.

### Antigen-specific CD4^+^ T cell response is compromised in mice infected with DLD deficient *L. major*

**Fig 7:**
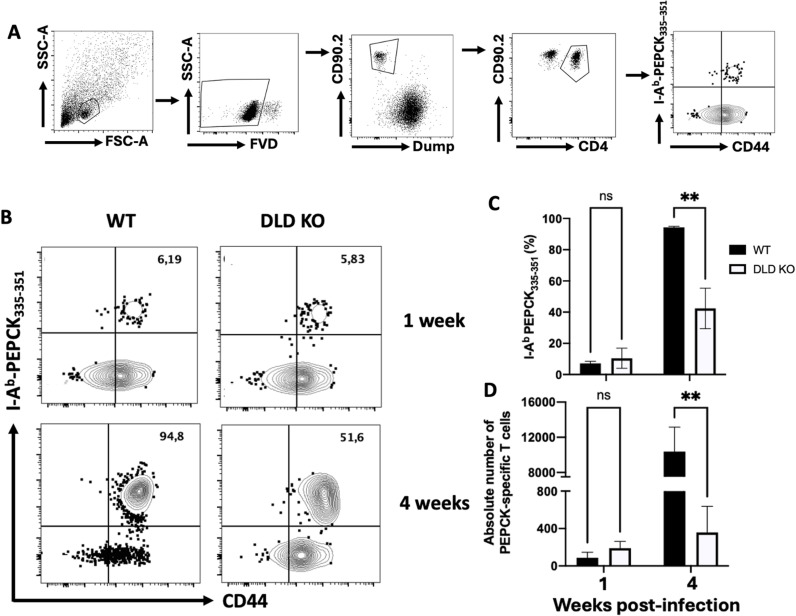
Impaired antigen-specific CD4 ^**+**^ **T cells response in mice infected with DLD deficient *L. major*.** C57BL/6 mice infected in the left hind footpad with 5 x10^6^ WT and DLD KO *L. major* were sacrificed at 1- and 4 weeks post-infection. Pooled suspensions of spleen and dLN cells were enriched for PEPCK-specific CD4^+^ T cells using I-A^b^-PEPCK_335-351_ tetramer and assessed by flow cytometry using the prescribed gating strategy (A). The flow plots show the percentages (B & C) and the absolute numbers (D) of PEPCK-specific CD4^+^ T cells were enumerated from the flow data. The results presented represent 2 independent sets of experiments (n = 4 mice per group) with similar results. *, p < 0.05, **; p < 0.01, ***; p < 0.001.

Previous studies have shown that reduced host immune cell recruitment during *Leishmania major* infections can result from reduced infectivity or proliferative properties of the mutant parasites [35,36]. To determine whether the blunted immune response in mice infected with DLD-deficient parasites is due to impaired parasite proliferation (resulting in lower antigen release) or DLD is immunodominance [12], we analyzed PEPCK-specific CD4^+^ T cell response (strongly induced after *L. major* infection) at different times after infection with WT and DLD deficient parasites by flow cytometry (Fig 7A). At one week (1 week) infection when parasite burdens were comparable, there was no significant difference in the frequency and absolute numbers of PEPCK-specific CD4^+^ T cells in spleens and dLN of mice infected with DLD deficient and WT parasites (Fig 7B-D). However, by four weeks post-infection when the numbers of DLD deficient parasites were significantly reduced (Fig 7B-D), the frequency and absolute numbers of PEPCK specific CD4^+^ T cells in spleens and dLNs of mice infected with DLD deficient *L. major* were markedly lower than in WT-infected mice (Fig 7B-D).

These results suggest that the diminished immune response in mice infected with DLD KO parasites is primarily due to their poor proliferation, which leads to reduced antigen expression. They also indicate that the blunted immune response in mice infected with DLD-deficient parasites is not attributable to the loss of DLD immunodominance which affects the overall host immune responses against the parasite.

### Vaccination with DLD deficient *L. major* confers protection against secondary virulent challenge

**Fig 8:**
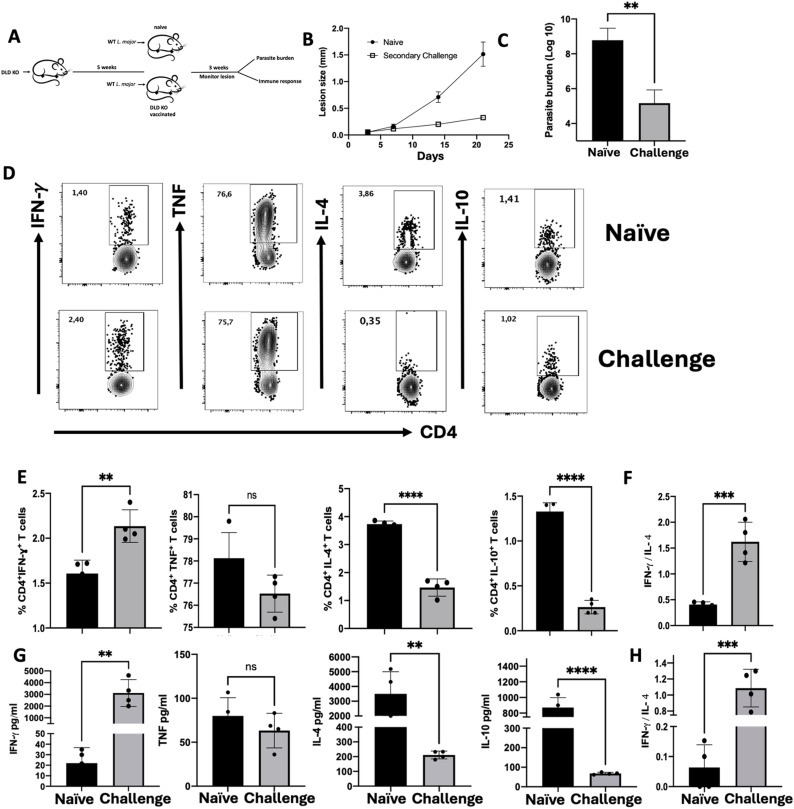
Vaccination with DLD deficient *L. major* in mice induces protection against virulent WT rechallenge. Six to eight weeks old Balb/c mice were injected in their right hind footpads with 2 x 10^6^ DLD deficient *L. major*. After 5 weeks, immunized mice and their age-matched naïve controls were challenged in the contralateral footpads with 1 x 10^5^ virulent *L. major* and lesion size was monitored weekly with digital calipers (A-Clip art source: https://openclipart.org/ & B). After 3 weeks, the challenged mice were sacrificed, and parasite burden was assessed by limiting dilution (C). Single cell suspensions from the dLNs were assessed for cytokine-producing CD4^+^ T cells directly *ex vivo* and assessed by flow cytometry. The flow plots and the frequency of the cytokine-producing CD4^+^ T cells were assessed (D & E). The ratio of the frequency of IFN-γ to IL-4-producing cells was also analyzed (F). Some cells were restimulated with SLA for 72 hours and the levels of cytokines (IL-4, IL-10, IFN-γ and TNF) in the culture supernatant fluids were assessed after 3 days by ELISA (G). The ratio of the IFN-γ to IL-4 secreted was analyzed (H). Results presented represent 2 independent sets of experiments with similar results, *, p < 0.05; **, p < 0.01; ***, p < 0.001; ns, not significant.

Durable immunity to cutaneous leishmaniasis is dependent on the presence of persisting parasites following recovery from primary infection [4,5,37,38]. Despite the significantly reduced IFN-γ immune response in mice infected with DLD deficient parasites (Fig 6), we hypothesized that their persistence without causing lesion could confer protection against virulent challenge, akin to findings in animals infected with *Leishmania* phosphoglycan deficient parasites [39]. To test this, we immunized mice via footpad infection with DLD KO parasites and challenged with WT parasites in their contralateral footpad after 5 weeks (Fig 8A). Immunized mice were protected as evidenced by significantly reduced lesion size and fewer parasite burden compared to their naïve age-matched controls (Fig 8B and 8C). This protection was associated with stronger immune response, as DLD deficient *L. major*-immunized mice displayed significantly higher proportion of IFN-γ-producing CD4^+^ T cells in their dLNs compared to naïve controls (Fig 8D and 8E). In contrast, frequencies of IL-10- and IL-4-producing CD4^+^ T cells were significantly reduced in immunized animals. In addition, the ratio of IFN-γ to IL-4 producing CD4^+^ T cells was significantly higher in the immunized animals compared to controls (Fig 8F). Similarly, dLN cell cultures from immunized mice secreted significantly higher levels of IFN-γ upon stimulation with SLA, while IL-10 and IL-4 levels were significantly lower by8 or 15 folds, respectively, compared to naïve mice (Fig 8G). Consistent with this, the ratio of the IFN-γ to IL-4 secretion was significantly increased in the immunized mice. (Fig 8H). Collectively, these findings demonstrate that vaccination with DLD KO *L. major* induces robust protective immune response against virulent WT *L. major* challenge.

## Discussion

Despite extensive research efforts, no approved vaccine currently exists against human leishmaniasis. Recovery from cutaneous leishmaniasis, the most prevalent disease form, is associated with durable and sometimes lifelong protection against reinfection [40], suggesting that prophylactic vaccination is possible. This natural infection-induced resistance forms basis for leishmanization, a practice historically used in many *Leishmania* endemic countries [41]. Natural or experimental infection-induced resistance is thought to be dependent on persistence of parasite persistence at the primary infection site, because sterile immunity does not confer long-term protection [5,37]. Consequently, a live-attenuated vaccine capable of maintaining persistent, non-pathogenic parasites is thought to be ideal vaccine candidate against cutaneous leishmaniasis [5,37,38,42]. Generating such a live-attenuated vaccine candidate requires a thorough understanding of the antigens that contribute to virulence and/or drive protective immune responses [12,43].

Using immunoproteomics approach, we identified that DLD_63-79_ peptide derived from *Leishmania major* DLD, an important metabolic enzyme in the parasite, as a strong inducer of CD4^+^ T cell response in a mouse model of experimental cutaneous leishmaniasis [12]. To fully understand the role of *L. major* DLD in immunopathogenesis of CL, we used CRISPR/Cas9 to delete the DLD gene. We found that the deletion greatly impaired the parasite’s proliferation *in vitro* (axenic culture) and caused dramatic alterations in mitochondrial properties. This impaired proliferation was recapitulated following *in vitro* and *in vivo* infections (in BMDMs and mice, respectively). The impaired proliferation of DLD-deficient parasites was evident in both *in vitro* macrophage and *in vivo* mouse infections. DLD-deficient parasites displayed impaired ability to induce lesions at the injection site and caused a dramatic reduction in the host immune response. The blunted immune response was not due to alteration in infectivity or loss of DLD as an immunodominant parasite antigen. Instead, it stemmed from impaired proliferation of DLD deficient parasites which limited the availability of parasite antigens necessary to stimulate robust host immune response.

In eukaryotic cells, DLD is a critical mitochondrial metabolic important for energy biogenesis [11]. Also known as dihydrolipoamide dehydrogenase, DLD is part of several enzyme complexes including pyruvate dehydrogenase, α-Ketoglutarate dehydrogenase, branched-chain amino acid dehydrogenase and glycine cleavage system (GCS) [25]. Within the GCS, DLD corresponds to the L protein subunit. There are 2 copies of the GCVL gene in *Leishmania*; *GCVL-1* and *GCVL-2,* which are located on chromosomes 29 and 32, respectively [11]. GCVL-2, the focus of our studies, has 25% sequence homology with GCVL-1 [11], and is catalytically more active than the GCVL-1 [11]. Both GCVL-1 and GCVL-2 act on the same substrate [11], suggesting they may be homologs [11]. Notably, we identified the peptide sequence, DLD_63-79_, that elicits strong protective immune response in mice infected with *Leishmania major*. This peptide is completely absent in GCVL-1 [12], highlighting the functional distinction between the two proteins. The observations reported in this study strongly suggest that deletion of *DLD* gene on chromosome 32 is responsible for the phenotype we observed in the DLD deficient parasites *in vitro* and *in vivo*. While GCVL-1 in *L. infantum* lacks signal sequences in their N-terminus and does not localize in the mitochondria [11,44], its localization and activity in *L. major* have not been studied. GCVL-2 DLD is present in secreted exogenous vesicles (exosomes) of *L. major* and plays a critical role in inducing IL-8 secretion in parasitized macrophages [45]. IL-8 is a critical pro-inflammatory cytokine produced by *Leishmania*-infected macrophages to recruit neutrophils to the site of infection [46]. This suggests that DLD in *Leishmania* modulates host immune responses during infection, which may be associated with the parasite’s virulence.

Several metabolic enzymes are essential for establishment and survival of *Leishmania* parasites in their parasitophorous vacuoles (PVs) niche in macrophages, their primary definitive host cell. These enzymes protect the parasites from the host cell toxic and degradative enzymes [47] and play a crucial role in maintaining the stringent metabolic balance necessary for amastigotes to survive in the PVs [8,9]. To adapt to this harsh niche, *Leishmania* parasites modulate their energy consumption and metabolic demands in the PVs [10,48,49]. For instance, phosphoenolpyruvate carboxykinase (PEPCK), a key gluconeogenesis enzyme, is expressed in the PV and supports the *de novo* synthesis of glucose [50], a scarce nutrient in this environment. As in this current study, our previous work demonstrated that PEPCK is critical for the intracellular survival and proliferation of *L. major* because PEPCK deficiency results in highly attenuated phenotype *in vitro* and *in vivo* [18]. Interestingly, deletion of other enzymes involved in essential metabolic pathways, such as gluconeogenesis [14], iron uptake [15], purine metabolism [16] and glycoconjugate synthesis [17], results in attenuated virulence. These mutants establish only low-level chronic infections without severe pathology, highlighting the importance of these enzymes for parasite survival and virulence. Taken together, these observations suggest that targeting metabolic enzymes critical for *Leishmania* survival inside macrophages could be a viable strategy for developing anti-leishmanial therapeutics.

DLD KO parasites were severely impaired in mitochondrial functions and ultrastructure and this was associated with impaired proliferation in axenic cultures. This is consistent with findings that mitochondrial respiration and function are intricately tied to parasite proliferation [29]. It is important to highlight that while oxygen consumption is essential for ROS production [51,52], mitochondrial ROS production and oxygen consumption can be independently modulated and are not entirely linked [53,54]. In addition, impaired mitochondrial ROS production has been linked to changes in mitochondrial ultrastructure [55,56]. Mitochondrial shape and network integrity are maintained by energy production during oxidative phosphorylation, which can be influenced by energy substrates or gene expression [57]. For instance, a change from oxidative phosphorylation to glycolytic metabolism result in thinning of mitochondrial membranes and increased tubule branching [58,59]. Similarly, genetic defects in fibroblasts has been shown to result in mitochondrial fragmentation and reduced energy production [60,61]. The loss of the cristae and membrane integrity observed in the DLD KO parasites suggests that their mitochondria are no longer capable of maintaining efficient respiration. This likely explains the compensatory increase in oxygen consumption observed in the functional assay. We acknowledge that our mitochondrial function experiments were conducted exclusively on promastigotes, and the observed impairments may not fully translate to the amastigote stage of the parasites. However, the impaired proliferation we observed in DLD KO parasites both inside macrophages *in vitro* (as amastigotes) and in infected mice strongly suggests that amastigotes *in vivo* (in mice) may also exhibit mitochondrial dysfunction. While the extent of mitochondrial impairment in amastigotes may differ from that observed in promastigotes, the phenotypic parallels *in vitro* and *in vivo* provide a basis for inferring similar mitochondrial deficiencies. Taken together, these observations highlight a critical relationship between mitochondrial metabolism and ultrastructure in *L. major*.

Similar to the current observation, the absence of PEPCK also leads to dysfunctional mitochondria, resulting to altered energy demands and impaired proliferation [18]. Interestingly, in *Trypanosoma brucei,* DLD plays different metabolic roles depending on the parasite stage. In the bloodstream form, which has limited mitochondrial function, DLD is crucial for thymidine biosynthesis necessary for DNA replication [22,62]. In contrast, in the procyclic form with fully functioning mitochondria, DLD deficiency disrupts 2-ketoglutarate dehydrogenase which is key component of the Krebs cycle [22,62]. In this study, we observed that DLD-deficient parasites (promastigotes), despite compromised mitochondrial function, may develop compensatory mechanisms to meet energy requirements. This is supported by elevated ECAR levels (Fig 8F), which is indicative of increased glycolytic activity [63]. However, these compensatory mechanisms appear insufficient for long-term survival. The long-term persistence of DLD deficient parasites may be due to the presence of GCVL-1 gene products that remain intact following GCVL-2 deletion (S1B and S1C Fig). Although GCVL-2 has a higher catalytic activity, both enzymes share similar substrates [11]. Future studies should explore the effects of simultaneous deletion of both GCVL-2 and GCVL-1 to fully understand the role of GCVL-1 in parasite persistence. This approach would also clarify the contribution of GCVL-1 to the survival and metabolic adaptability of DLD-deficient *Leishmania* parasites.

Although multiple factors influence resistance to cutaneous leishmaniasis, the induction of IFN-γ producing CD4^+^ T cells (Th1 cells) is the critical component that ensures effective parasite killing in infected macrophages [4,5]. Previous studies have shown that the absence of key *Leishmania* surface molecules essential for parasites survival can impair the optimal development of Th1 immune response [39]. Indeed, we recently demonstrated that deficiency of PEPCK in *L. major* induced a poor Th1 immune response [18]. In the current study, we found that DLD KO parasites induced a blunted immune response in infected animals (Fig 5). Despite this, mice immunized with these parasites were strongly protected against a subsequent challenge with virulent wild-type parasites (Fig 7B and 7C). This suggests that DLD-deficient parasites may be a promising live-attenuated vaccine candidate. Nevertheless, the use of a live-attenuated vaccine is still controversial due to concerns about potential reactivation of virulence and disease recrudescence in immunocompromised individuals [41]. Previous reports have consistently shown that vaccination with heat-killed or irradiated parasites fails to induce long-term protection equivalent to that conferred by live-attenuated parasites [64–68]. The protection DLD KO immunized mice was associated with increased IFN-γ production and a concomitant reduction in disease-exacerbating (IL-4 and IL-10) cytokines. Thus, while primary infection with DLD-deficient parasites elicited a blunted immune response, it effectively primed the immune system for robust secondary recall response able to protect against subsequent challenge.

In conclusion, this study provides the first evidence of the critical role of DLD in virulence and immunopathogenesis of *Leishmania major*. We showed that DLD deficiency leads to impaired mitochondrial function, reduced parasite proliferation *in vitro,* loss of pathogenicity *in vivo* and dramatically blunted host immune response. Notably, DLD-deficient parasites were able to persist in immunized mice and conferred robust protection against virulent rechallenge. While it is important to investigate whether DLD-deficient parasites could regain virulence after prolonged persistence as was observed for lipophosphoglycan 2 (lpg2) deficient parasites [69,70], the strong protective immunity elicited in immunized animals suggests that DLD deficient parasites hold promise as a live-attenuated vaccine candidate against cutaneous leishmaniasis.

## Materials and Methods

### Ethics statement

This study was approved by the University of Manitoba Animal Care Committee Protocol Number 21-022. The experiments involving mice were carried out according to the regulation and recommendation of the Canadian Council on Animal Care.

### Mice

Six to eight-week-old female C57BL/B6J mice were obtained from the Genetic Modeling Centre (GMC) at the University of Manitoba. In some experiments, 6–8-week-old female Balb/c mice were obtained from Charles River (Senneville, Quebec, Canada). Mice were housed at the Central Animal Care Services facility at the University of Manitoba.

### Parasites preparation

*Leishmania major (L. major)* Fredlin (MHOM/80/Fredlin) strain was used for the entire study. Promastigotes were cultured at 26 °C in M199 medium (Hyclone, Logan, UT) supplemented with 20% heat-inactivated fetal bovine serum (FBS) (Cansera, Mississauga, ON, Canada), and 100 U/ml penicillin/streptomycin. Stationary phase-promastigotes from day 7 cultures were washed three times with sterile phosphate-buffered saline (PBS). Depending on the experiments, a defined number of parasites was injected subcutaneously into the left hind footpad or used to infect bone marrow derived macrophages.

### Generation of *DLD* gene-deficient *L. major* and complementary Add back control *L. major*

To generate DLD deficient *L. major,* we used the CRISPR-Cas9, which has been demonstrated to efficiently delete multicopy and essential genes of *Leishmania* [33]. There is a single copy of *GCVL-2 DLD* gene in *Leishmania* species on chromosomes 32 (Fig 1). Briefly, an all-in-one plasmid (pLDCN) containing two short oligonucleotide sequences (guide RNA; gRNA) complementary to the targeted *DLD* gene is introduced into logarithmic cultures of *L. major* such that upon expression within the parasites, a ribonucleocomplex is formed with Cas9 to initiate cleavage. Following cleavage and subsequent maintenance in, G418, a homology-directed repair mechanism was initiated by introducing a donor DNA (bleomycin) PCR product by electroporation. This was followed by serial maintenance in a medium containing Phleomycin. Once stable cultures were generated, *DLD* gene null mutants were selected by limiting dilution to screen for single parasite clones. To generate a complementary (add back) control strain of *L. major*, we reintroduced the *DLD* gene episomally into the DLD deficient *L. major* parasites as previous described [33]. Gene constructs containing *DLD* were cloned into the BamH1 sites of the A2-Rel amastigote specific cluster of the *Leishmania* specific plasmid, pLPHyg, as described previously [71]. The resulting construct, pLPHyg-DLD was introduced to the logarithmic phase DLD deficient *L. major* parasites by electroporation. Successful generation of DLD deficient parasites and complementary addback control was confirmed both by traditional PCR (for the absence of *GCVL-2 DLD* gene product) and by RT-qPCR for DLD mRNA expression after normalization with r18SRNA. The DLD primers used for PCR are F-5’ GTCCTACGACGTGACGGTGA R-3’ TTCTCGTCGAAGGGCAGGAA with cycling parameters as follows; Denaturation; 94 °C for 5minutes, Annealing; 65 °C for 20 seconds and Extension; 72 °C for 30 seconds repeated for 30 cycles. The *DLD* gene deletion and generation of addback control was further confirmed *in vivo* by assessing the frequency of DLD-specific CD4^+^ T cells in the spleens of mice infected with wild-type, DLD deficient and complementary addback parasites using DLD tetramer as previously described [12]. The expression of GCVL-1, a homolog of DLD, in DLD KO parasites was assessed by PCR and RT-qPCR using the following primer pairs: F (5′-TGTCACGAAGAAAGACGGCA) and R (5′-ATGCTACTGCTCTCCATCGC). The cycling parameters were the same as those used for GCVL-2.

### Western blotting assay

Logarithmic-phase promastigotes (final concentration: 1 x 10⁶/mL) were harvested and lysed in 2x Laemmli buffer containing 5% 2-mercaptoethanol (Sigma-Aldrich, St. Louis, MO). The lysates were heated at 95 °C for 5 minutes, and the soluble fractions were loaded onto a 12% SDS-PAGE gel. Protein separation was performed at 100 V for 1.5 hours and transferred onto an activated PVDF membrane at 20 V for 1 hour using a semi-dry western transfer system (Bio-Rad, Mississauga, ON, Canada). The membrane was immersed in tris-buffered saline (TBS) containing 0.5% Tween-20 and 5% non-fat milk (pH 7.4) for 1 hour at room temperature to block nonspecific binding. Subsequently, the membrane was incubated overnight at 4 °C with a polyclonal rabbit anti-DLD antibody (1:1000 dilution) prepared in blocking solution (TBS supplemented with 3% BSA). After three washes with TBS containing 0.2% Tween-20 (TBS-T), each lasting 15 minutes, the membrane was incubated for 1 hour at room temperature with a horseradish peroxidase-conjugated mouse anti-rabbit secondary antibody (1:10,000 dilution) in blocking solution. Following three additional washes with TBS-T (30 minutes per wash), protein bands were visualized using the ECL detection reagent (GE Healthcare, Mississauga, ON, Canada) and imaged using a Bio-Rad ChemiDoc Imaging System.

### Assessment of growth characteristics and mitochondrial function in DLD deficient (knock-out, KO) parasite in axenic cultures

Equal numbers of 3-day promastigotes of wild-type (WT), DLD KO and DLD AB *L. major* were grown in axenic culture and counted daily under an optical microscope using a hemocytometer. In some experiments, the DLD expression from the kinetics of DLD AB parasites growing in the presence/absence of hygromycin were assessed overtime by PCR. The mitochondrial function (mitochondrial membrane potential and mitochondrial reactive oxygen production) of logarithmic growing parasite lines was measured by using Tetramethylrhodamine, Methyl Ester, (TMRM) and mitosox dyes (Thermofisher scientific) based on our previous established protocol [18]. TMRM and mitosox stained parasites were gated in the PE and PI channels using the Cytoflex flow cytometer. The mean fluorescent intensity was assessed using Flow jo software (v10).

### Mitochondrial respiration of the parasite lines

Mitochondrial respiration in the different parasite lines was assessed in a mitostress test using a Seahorse analyzer as previously described [30]. Briefly, 1ml of poly D lysine was incubated in the wells of an XF24 analyzer plate for 2 hours at room temperature. Five million parasites each of WT, DLD KO and DLD AB were immobilized in the treated XF24 analyzer plate and 100 μl of prewarmed (37 °C) XF DMEM media supplemented with 1 mM Sodium Pyruvate and 11 mM D-glucose were added. The plates were centrifuged at 3000 rpm and for 10 min and left in a 37 °C incubator for 1hr. Drugs (oligomycin, carbonyl cyanide-4-(trifluoromethoxy) phenylhydrazone (FCCP), and rotenone/antimycin A) up to a final concentration of 1 mM were added to the appropriate wells of the pre-warmed plate. Afterwards, the plates were put into the XF24 analyzer machine and calibrated. After incubation of the XF plates containing the immobilized parasites, they were topped up with 400 μl of XF complete media and mitostress analysis was performed using the Seahorse analyzer machine to measure oxygen consumption rate (pmol/min). Thereafter, the parasites were lysed and total protein concentration was assessed using the Bradford Assay. The results were normalized using the Agilent software as oxygen consumption rate per protein concentration (pmol/min/μg/ml).

### Visualizing mitochondrial ultrastructure of the parasites

The mitochondrial ultrastructure of the parasite lines were visualized by transmission electron microscopy (TEM) using protocols recently described [72]. Briefly, 10ml of logarithmic phased parasite lines were spun in a 15ml Falcon tube at 3000rpm for 7 min. Pellets were resuspended in PBS by pipetting up and down. Following centrifugation at 3000rpm for 7 min, pellets were fixed in 1ml of fixation solution (0.1M Sorensen’s phosphate buffer and 3% glutaraldehyde) for 3hrs at RT. Afterwards, fixation solution were removed by centrifugation at 3000rpm for 7 min and pellets were resuspended in 1ml of sucrose solution (5% sucrose in 0.1M Sorensen’s Phosphate Buffer). Fixed parasites were embedded onto the stage of a transmission electron microscope and a trained histologist identified the mitochondrial ultrastructure of the parasite lines from images captured at 34000x magnification. Images were analyzed using the Field-Based Assessment approach, which involved evaluating multiple fields across all samples to quantify mitochondrial damage. Damaged mitochondria were characterized by disrupted double membranes and disorganized cristae.

### Bone marrow-derived macrophage preparation and infection

Bone marrow-derived macrophages (BMDMs) were obtained from bone marrow cells as described by Ikeogu et al [73]. Briefly, bone marrow cells (4 x 10^6^/ml) were plated in Petri dishes in complete medium (RPMI 1640 medium supplemented with 10% heat-inactivated fetal bovine serum [FBS], and 100 U/ml penicillin/streptomycin, 5 x 10^-5^ 2-ME, and 2 mM L-glutamine) in the presence of 30% L929 cell supernatant. After 3 days, cells are given an additional complete RPMI supplemented with 30% L929 cell supernatant. On day 7, differentiated BMDMs were gently scrapped out of the petri dishes, washed with complete medium, resuspended in complete medium at 1 x 10^6^/ml and assessed for purity (relative expression of F4/80 molecule) by flow cytometry.

For infection, 500 μl of purified BMDMs in complete RPMI media were seeded in 5 ml polypropylene tubes and infected with different parasite lines to a final parasite:BMDM ratio of 10:1. After 6 hrs, free parasites were washed off with 2 ml sterile PBS by centrifugation at 500 rpm. Afterwards, 500 μl of complete medium was added to each tube and incubated at 27 °C. At 24, 48 and 72 hr post-infection, infection rate and number of parasites per infected cell were monitored by staining cytospin preparations with Giemsa and assessing with an optical microscope.

### Mice infection and challenge studies

For primary infections, female BALB/c or C57BL/6 mice (8-10 weeks old) were infected in their right hind footpads with 1 x 10^6^ (WT and DLD AB) or 1 x 10^6^, 5 x 10^6^ or 10 x10^6^ (DLD KO depending on the study) parasites. The uninfected footpads were injected with sterile PBS to serve as control. For challenge studies, some mice infected with DLD KO parasites and age-matched naïve controls were infected in their non- immunized footpads (left footpad) with 1 x 10^5^ WT parasites. Challenged mice were assessed weekly for lesion development and sacrificed at the indicated times to determine parasite burden and measure cytokine response in the spleens and draining lymph nodes.

### Measurement lesion size and estimation of parasite burden

The footpads of infected mice were assessed for lesion developments weekly using digital calipers and lesion size was calculated as the difference between infected and contralateral uninfected footpads. In the challenge experiments, lesion size was calculated as the difference between the immunized and unimmunized footpads. At the specified time points, infected mice were sacrificed and parasite burden in the footpads was assessed by limiting dilution as described by Titus et al [74].

### Cytokine production measurements

At the specified points, infected mice were euthanized and single cell suspensions of the lymph nodes draining the infection sites (dLNs) and spleens were prepared and stimulated with soluble *Leishmania* antigen (SLA, 50 μg/ml). After 3 days, the level of cytokines secreted into the culture supernatant fluids was measured using the multiplex cytokine profiling 10-plex assay (Eve technologies, Calgary Alberta).

### Intracellular cytokine measurement and tetramer staining

For direct *ex vivo* cytokine measurement, single-cell suspensions of spleens and dLNs (1 ml) were stimulated with a 2 μl of cell stimulation cocktail (containing PMA/ionomycin and Brefeldin A, Biolegend) for 4 hours at 37 ˚C. Thereafter, the cells were surface-stained, fixed and permeabilized with a Cytofast fix/perm solution (Biolegend) for 20 minutes at room temperature. Thereafter, the cells were washed with 1x Cytofast perm wash buffer (Biolegend) for 5 minutes and stained for 20 minutes at room temperature with previously determined optimal concentration of fluorochrome-conjugated antibodies against IL-4, IFN-γ and TNF-α in 100 μl of 1x Cytofast perm wash buffer. Stained cells were washed with FACS buffer, acquired on the FACS Canto II flow cytometer (BD Bioscience, Mississauga, ON, Canada), and analyzed using FlowJo software (TreeStar, Ashford, OR).

In some experiments, the frequency of *Leishmania* DLD- or PEPCK-specific CD4^+^ T cells in the infected mice were assessed using Tetramer enrichment staining protocol as previously described by Moon et al [75]. Briefly, single cell suspension from the peripheral lymph nodes (LNs) and spleens of C57BL/6 mice infected with WT, DLD KO or DLD AB parasites were stained with allophycocyanin-labeled PEPCK_335-351_ or DLD_63-79_ pMHC tetramer to a final concentration of 10 nM for 30 minutes at 37 ˚C. Following washing with cold cell sorting buffer (2% FBS and 1 mM EDTA in PBS), PEPCK or DLD specific tetramer-labeled cells were resuspended in 200 μl cell sorting buffer and incubated with 50 μl of anti-allophycocyanin–conjugated magnetic microbeads (Miltenyi Biotec) for 30 minutes at 4 ˚C. Thereafter, tetramer-bound cells were enriched by passing the cells through Miltenyi LS column held by a magnet. Enriched tetramer-bound cells were surfaced stained with other fluorochrome conjugated antibodies such as anti-CD4, CD44, CD90.2 CD19, F480, CD11b and FVD, washed, acquired using a Cytoflex flow cytometer and analyzed using the Flowjo software.

### Assessment of metacyclogenesis

Metacyclogenesis was assessed using fluorochrome-conjugated peanut lectin agglutinin (PNA-FITC) as previously described [76]. Briefly, 5 x 10^6^ logarithmic or stationary phase WT, DLD KO or DLD AB parasites were washed in PBS and stained with 50 μg/ml of PNA-FITC in complete DMEM. Following incubation for 30 minutes at RT in the dark, stained parasites we washed in PBS and PNA signals detected using the Cytoflex flow cytometer and analyzed using Flowjo software.

### Statistical analysis

Data are presented as mean ± SEM. To compare two groups, a two-tailed Student t test was used. One-way or two-way ANOVA was used to compare multiple (> two) groups. Differences between two or more groups are considered statistically significant if the p value was less than 0.05 (p < 0.05).

## Supporting information

S1 Fig GCVL-1 DLD expression in DLD KO parasites. Assessment of protein expression of GCVL-2 DLD by western blots using polyclonal anti-DLD primary antibody in wild type (WT), DLD KO and DLD KO addback (DLD AB) parasites (A). Detection of GCVL-1 DLD gene product (357 bp) by PCR (B), and GCVL-1 DLD mRNA expression by RT-PCR (C) in DLD KO parasites. ns, not significant.
(DOCX)

S2 Fig Loss of DLD GCVL-2 expression in the absence of hygromycin (Hyg). The pLPHyg-DLD plasmid was introduced into DLD-deficient L. major parasites in the logarithmic growth phase via electroporation. Transfected parasites were cultured in axenic media with (+) or without (-) hygromycin. At the indicated time points post-culture, the presence of the GCVL-2 DLD gene product (A) and its mRNA expression (B) was analyzed using PCR and RT-PCR, respectively. **, p <0.01; ****, p < 0.0001; ns, not significant.
(DOCX)

S3 Fig **Dose-dependent induction of disease pathology by DLD KO parasites.** Six- to eight-week-old Balb/c mice (n = 6-8 per group) were infected in the left hind footpad with either 1×10^6^ wild-type (WT) parasites or 5–10 (1-10 x 10^6^) times higher doses of DLD knockout (KO) parasites. Lesion development was monitored weekly using digital calipers (A). At 4 weeks post-infection, the mice were sacrificed, and parasite burden was quantified by limiting dilution (B). **, p < 0.01; ****, p < 0.0001.
(DOCX)

S1 Data Minimal data sets for Figs 1–8 and S1–S3. (XLSX)
