# Supplementary material for: Leishmania major Dihydrolipoyl dehydrogenase (DLD) is a key metabolic enzyme that drives parasite proliferation, pathology and host immune response
Source: PLoS Pathog. 2025 Mar 17;21(3):e1012978. doi: 10.1371/journal.ppat.1012978 (PMC11949353; doi:10.1371/journal.ppat.1012978)
Supplement: S1 Fig — GCVL-1 DLD expression in DLD KO parasites. Assessment of protein expression of GCVL-2 DLD by western blots using polyclonal anti-DLD primary antibody in wild type (WT), DLD KO and DLD KO addback (DLD AB) parasites (A). Detection of GCVL-1 DLD gene product (357 bp) by PCR (B), and GCVL-1 DLD mRNA expression by RT-PCR (C) in DLD KO parasites. ns, not significant. (DOCX) [file ppat.1012978.s001.docx]

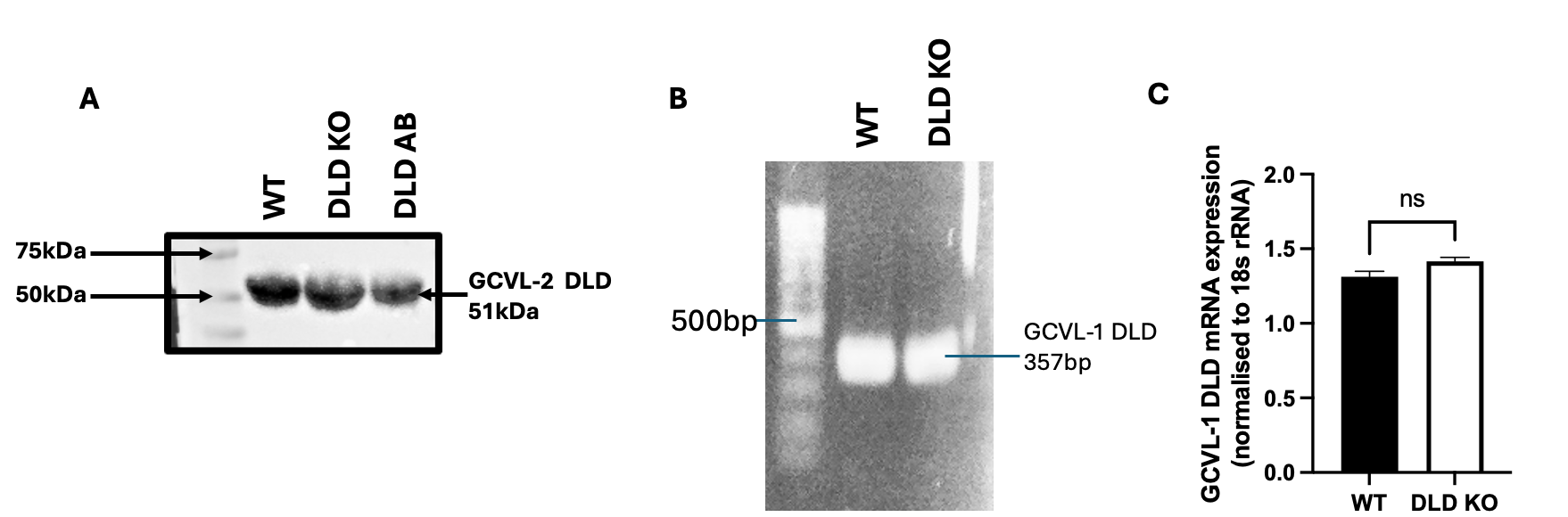


**S1 Fig: GCVL-1 DLD expression in DLD KO parasites.** Assessment of protein expression of GCVL-2 DLD by western blots using polyclonal anti-DLD primary antibody in wild type (WT), DLD KO and DLD KO addback (DLD AB) parasites (A). Detection of GCVL-1 DLD gene product (357bp) by PCR (B), and GCVL-1 DLD mRNA expression by RT-PCR (C) in DLD KO parasites. ns, not significant.
