# Supplementary material for: Leishmania major Dihydrolipoyl dehydrogenase (DLD) is a key metabolic enzyme that drives parasite proliferation, pathology and host immune response
Source: PLoS Pathog. 2025 Mar 17;21(3):e1012978. doi: 10.1371/journal.ppat.1012978 (PMC11949353; doi:10.1371/journal.ppat.1012978)
Supplement: S2 Fig — Loss of DLD GCVL-2 expression in the absence of hygromycin (Hyg). The pLPHyg-DLD plasmid was introduced into DLD-deficient L. major parasites in the logarithmic growth phase via electroporation. Transfected parasites were cultured in axenic media with (+) or without (-) hygromycin. At the indicated time points post-culture, the presence of the GCVL-2 DLD gene product (A) and its mRNA expression (B) was analyzed using PCR and RT-PCR, respectively. **, p <0.01; ****, p < 0.0001; ns, not significant. (DOCX) [file ppat.1012978.s002.docx]

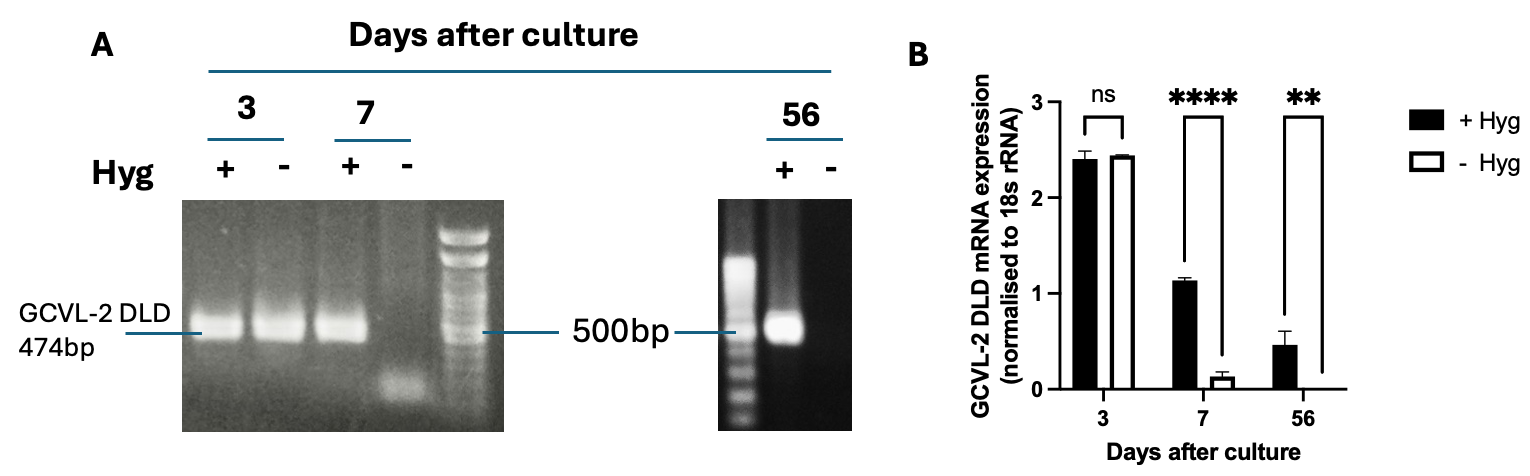


**S2 Fig: Loss of DLD GCVL-2 expression in the absence of hygromycin (Hyg).** The pLPHyg-DLD plasmid was introduced into DLD-deficient L. major parasites in the logarithmic growth phase via electroporation. Transfected parasites were cultured in axenic media with (+) or without (-) hygromycin. At the indicated time points post-culture, the presence of the GCVL-2 DLD gene product (A) and its mRNA expression (B) was analyzed using PCR and RT-PCR, respectively. **, p <0.01; ****, p < 0.0001; ns, not significant.
