# Supplementary material for: Leishmania major Dihydrolipoyl dehydrogenase (DLD) is a key metabolic enzyme that drives parasite proliferation, pathology and host immune response
Source: PLoS Pathog. 2025 Mar 17;21(3):e1012978. doi: 10.1371/journal.ppat.1012978 (PMC11949353; doi:10.1371/journal.ppat.1012978)
Supplement: S3 Fig — Dose-dependent induction of disease pathology by DLD KO parasites. Six- to eight-week-old Balb/c mice (n = 6-8 per group) were infected in the left hind footpad with either 1×10^6^ wild-type (WT) parasites or 5–10 (1-10 x 106) times higher doses of DLD knockout (KO) parasites. Lesion development was monitored weekly using digital calipers (A). At 4 weeks post-infection, the mice were sacrificed, and parasite burden was quantified by limiting dilution (B). **, p < 0.01; ****, p < 0.0001. (DOCX) [file ppat.1012978.s003.docx]

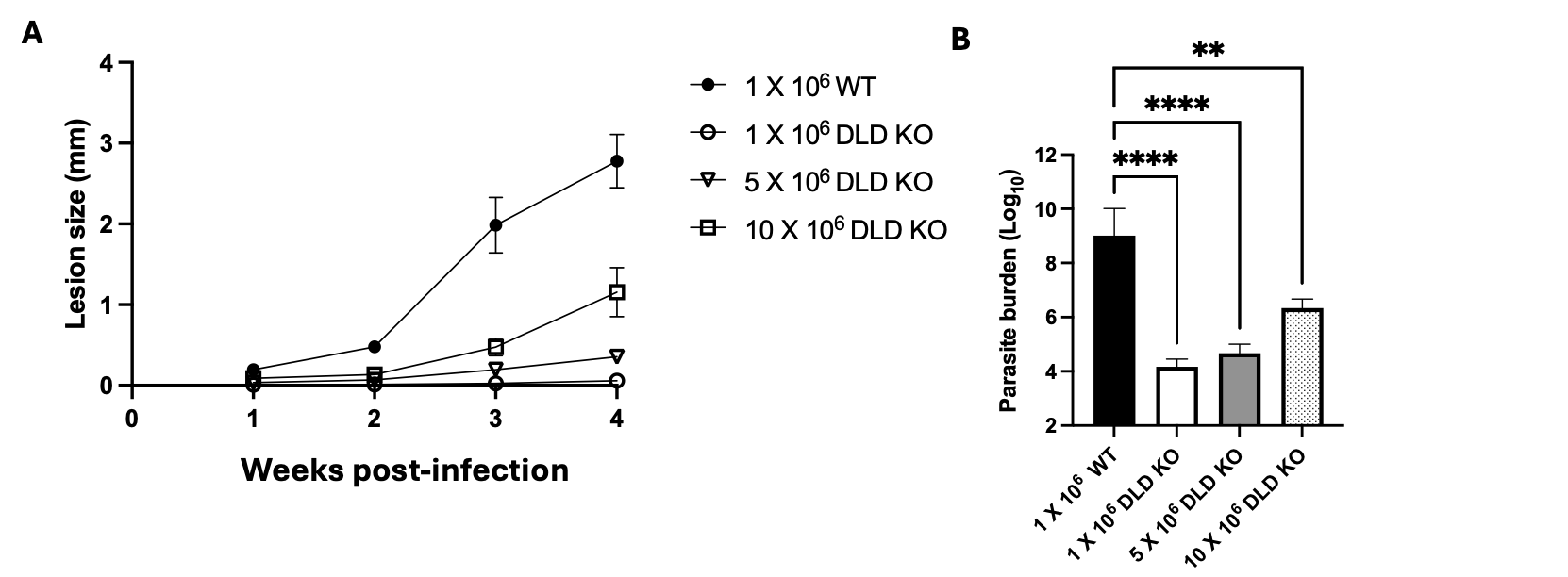


**S3 Fig: Dose-dependent induction of disease pathology by DLD KO parasites.**
Six- to eight-week-old Balb/c mice (n = 6-8 per group) were infected in the left hind footpad with either 1×10^6^ wild-type (WT) parasites or 5–10 (1-10 x 10^6^) times higher doses of DLD knockout (KO) parasites. Lesion development was monitored weekly using digital calipers (A). At 4 weeks post-infection, the mice were sacrificed, and parasite burden was quantified by limiting dilution (B). **, p < 0.01; ****, p < 0.0001
